# Effects of autonomy-supportive ski instruction on skill acquisition, mental toughness, and self-efficacy in novice skiers: a cluster-randomized controlled trial

**DOI:** 10.3389/fpsyg.2026.1828839

**Published:** 2026-05-29

**Authors:** Huanyu Gao, Dawei Cao, Yangyang Ren, Hongbo Shi, Yuxiang Liang, Yuanguo Liu

**Affiliations:** 1 School of Ice and Snow Sports, Shenyang Sport University, Shenyang, China; 2 School of Physical Education, Huaibei Normal University, Huaibei, China; 3 Graduate School, Harbin Sport University, Harbin, China

**Keywords:** autonomy-supportive teaching, mental toughness, self-determination theory, self-efficacy, ski instruction, skill acquisition

## Abstract

**Background:**

Instructional interactions in beginner skiing classes may influence both objective skill performance and psychological outcomes. However, longitudinal intervention evidence in high-risk skill-learning contexts remains limited, and objective skill performance and psychological outcomes have often been examined separately.

**Objective:**

This study aimed to compare the effects of autonomy-supportive teaching and conventional teaching on skill acquisition, self-efficacy, and mental toughness in beginner skiing instruction.

**Methods:**

A cluster-randomized controlled trial was conducted with 216 novice skiers from eight intact classes. The instructional program lasted 3 weeks and followed an intensive short-term schedule, with three 200-min sessions per week. Skill assessments and questionnaires were administered at three time points: after the standardized teaching phase in Week 1, after the Week 2 instructional sessions, and after the Week 3 instructional sessions. Linear mixed-effects models were used to examine Group × Time interaction effects.

**Results:**

Learners receiving autonomy-supportive teaching reported significantly greater increases in perceived teacher autonomy support than those receiving conventional teaching. Skill acquisition, self-efficacy, and mental toughness all showed significant Group × Time interaction effects, with the autonomy-supportive teaching group demonstrating greater incremental improvements over time.

**Conclusion:**

Autonomy-supportive teaching can simultaneously improve skill acquisition, self-efficacy, and mental toughness among novice skiers without increasing instructional time or practice volume. This study provides longitudinal empirical evidence for evaluating pedagogical intervention effects in high-risk skill-learning contexts.

## 1 Introduction

China has witnessed sustained growth in ice and snow sports participation in recent years, with skiing progressively incorporated into school physical education curricula and broader training systems as a key avenue for promoting adolescents’ physical fitness and sport engagement (Song and Cui, 2025). Compared with conventional sports, however, skiing combines elevated risk with substantial technical demands. Learners must maintain postural control and movement coordination under conditions of low temperature, variable slopes, and complex terrain—demands that impose considerable requirements on risk awareness and risk-coping capacity (Çağın et al., 2024). For beginners, slow technical progress, frequent falls, and fear-related experiences often trigger frustration and avoidance, thereby undermining learning motivation and increasing dropout rates (Cigrovski et al., 2018). Despite the practical need to synergistically optimize safety management, skill progression, and positive psychological experiences, high-quality causal evidence addressing these interconnected outcomes remains limited.

Self-Determination Theory (SDT) provides a robust explanatory framework for understanding motivational and behavioral variability in beginner skiing instruction. SDT posits that satisfaction of the basic psychological needs for autonomy, competence, and relatedness fosters intrinsic motivation, sustained engagement, and adaptive emotional experiences (Deci and Ryan, 2000; Ryan and Deci, 2020, 2024). When these psychological needs are supported, learners are more likely to sustain goal-directed practice in the face of difficulty and performance setbacks; conversely, need frustration predisposes learners to heightened anxiety, frustration, and avoidance tendencies (Ma, 2021; Fierro-Suero et al., 2024). Instructional interaction style thus constitutes a critical determinant of learning process quality.

Autonomy-supportive teaching (AST), a central pedagogical application of SDT, encompasses practices such as offering meaningful choice, providing rationales for task value, acknowledging learners’ perspectives, responding constructively to negative emotions, employing non-controlling language, and respecting individual learning pace (Patall et al., 2010; Jang et al., 2016; Steingut et al., 2017; Reeve and Cheon, 2021; Cheon et al., 2023). Accumulated evidence and systematic reviews have consistently documented positive associations between AST and learning motivation, classroom engagement, emotional experiences, psychological need satisfaction, and learning outcomes (Bureau et al., 2022; Reeve and Cheon, 2024; Patzak and Zhang, 2025). Beginner skiing provides a more challenging instructional context for testing AST. Novice skiers must learn motor control under conditions involving changes in speed, slope variation, and risk of falling. In this context, teachers’ task explanations, feedback style, and responses to learners’ emotions may directly influence whether learners continue to practice, how they interpret failure feedback, and whether they remain engaged under tension and frustration. For this reason, beginner skiing provides a suitable context for examining the applicability of autonomy-supportive teaching in high-risk skill learning. However, few studies have integrated randomized controlled designs, skill acquisition assessment, and psychological resource measures, making it difficult to determine the overall intervention effect of autonomy-supportive teaching in beginner skiing instruction.

Within beginner skiing, self-efficacy and mental toughness hold particular explanatory relevance. Self-efficacy—defined as individuals’ beliefs in their capabilities to execute courses of action required to attain designated performance levels—is robustly associated with effort investment and persistence (Bandura, 1982; Al-Abyadh and Abdel Azeem, 2022; Clemente et al., 2024), with snow sport research further indicating its relevance to performance and emotional experience (Cigrovski et al., 2018; Sklett et al., 2018). Mental toughness, conceptualized as the capacity to maintain functional stability and performance levels under pressure and challenge, relates to training quality, sustained engagement, and athletic performance (Jones et al., 2002; Gucciardi et al., 2015; Cowden, 2017; Hsieh et al., 2024). Within the SDT framework, AST may foster these psychological resources by supporting competence experiences, providing informational feedback, and optimizing classroom interactions, thereby establishing more favorable psychological conditions for skill acquisition (Ma, 2021; Hsieh et al., 2024).

Taken together, the preceding discussion indicates that three issues require further clarification: whether the effects of autonomy-supportive teaching in beginner skiing are supported by randomized controlled trial evidence; whether autonomy-supportive teaching can simultaneously influence skill acquisition and psychological outcomes within the same intervention framework; and whether existing evidence can be extended to high-risk skill-learning contexts such as skiing. To address these issues, the present study employed a cluster-randomized controlled trial in beginner skiing instruction, using intact classes as clusters, to compare the effects of autonomy-supportive teaching and conventional teaching on skill acquisition, self-efficacy, and mental toughness across three time points (T0, T1, and T2). A manipulation check was conducted to examine whether the two instructional conditions produced distinct teaching styles. The following hypotheses were tested:

*H1*: Autonomy-supportive teaching would enhance skill acquisition among novice skiers.

*H2*: Autonomy-supportive teaching would increase self-efficacy among novice skiers.

*H3*: Autonomy-supportive teaching would strengthen mental toughness among novice skiers.

The findings may provide more causally informative evidence for optimizing pedagogy in high-risk skill-learning contexts.

## 2 Methods

### 2.1 Study design

This study employed a cluster-randomized controlled trial design, with intact classes serving as clusters. A total of eight intact classes were included and randomly assigned to either the experimental group (four clusters) or the control group (four clusters). The instructional program lasted 3 weeks and comprised three 200-min sessions per week, for a total of nine sessions.

The program consisted of a 1-week standardized teaching phase followed by a 2-week differential intervention phase. During the standardized phase, both groups received identical safety instruction, including ski slope safety rules and fall-protection techniques, as well as foundational technical instruction, including flat-ground exercises and basic stance training. This phase was designed to standardize risk-control procedures and reduce the feasibility problems associated with assessing true beginners before any formal exposure.

The differential intervention was implemented during Weeks 2 and 3. The experimental group received autonomy-supportive teaching, whereas the control group continued with conventional teaching. Outcome assessments, including skill evaluations and questionnaires, were conducted at three time points: T0, T1, and T2. T0 was completed at the end of Week 1, after the standardized teaching phase and before the differential intervention began. T1 and T2 were completed after the instructional sessions in Weeks 2 and 3, respectively. The reporting of this study was guided by the CONSORT extension for cluster randomized trials, with attention to the unit of randomization, number of clusters, allocation procedure, participant flow, handling of clustering effects, and multilevel statistical analysis.

### 2.2 Participants

Participants were first-year undergraduate students from Shenyang Sport University who were not majoring in skiing. They were drawn from eight intact classes, with approximately 24–28 students in each class. Before the course began, none of the participants had received systematic ski training, and any previous skiing experience was limited to no more than one introductory or recreational exposure. All participants were in good physical health and had no history of musculoskeletal disorders affecting lower-limb force control or balance.

Inclusion and exclusion criteria were established to ensure sample appropriateness. The inclusion criteria were as follows: (1) ability to participate normally in course learning and practice; (2) no prior history of systematic ski training; and (3) provision of informed consent. The exclusion criteria were: (1) recent sports injury or a history of cardiovascular, vestibular, or neurological disorders; and (2) marked fear or avoidance responses that made participation in basic instructional tasks difficult, as jointly confirmed by the instructor and research staff. Students who met the exclusion criteria were allowed to continue participating in routine instruction but were not included in the statistical analyses. During the study, participants were classified as lost to follow-up if they missed two consecutive sessions, were unable to complete assessments because of injury or illness, or withdrew voluntarily. Baseline information for these participants was retained, but they were excluded from subsequent repeated-measures analyses. The final analytic sample was defined as the number of participants who completed all assessments from T0 to T2.

Sample size estimation was performed using G*Power 3.1, with skill acquisition specified as the primary outcome. A medium effect size of *d* = 0.50, statistical power of 1 − *β* = 0.80, and a significance level of *α* = 0.05 were specified. Because classes were used as the unit of randomization, observations from students within the same class could not be treated as fully independent; therefore, the sample size estimation was adjusted for class-level clustering. As no directly comparable ICC estimate was available for beginner skiing instruction, a design effect of 1.20 was used as the clustering adjustment parameter in the *a priori* sample size planning. After further accounting for an anticipated attrition rate of 10%, the minimum required sample size was estimated to be 192 participants. A total of 216 participants completed all assessments from T0 to T2, meeting the planned sample size requirement. To improve the transparency of cluster reporting, baseline class-level ICCs for the main outcomes were reported in the Results section, and random intercepts for both class and student were specified in the linear mixed-effects models to account for class-level clustering and repeated measurements.

### 2.3 Randomization and blinding

Randomization was conducted at the class level. Before the study began, a research team member who was not involved in teaching or assessment generated the random allocation sequence using computer-generated random numbers, assigned the eight intact classes to four experimental clusters and four control clusters, and sealed the allocation results. To minimize expectancy effects and differential instructional cues during the standardized teaching phase, group allocation was disclosed to the instructors and research assistants only after completion of the T0 assessments. The differential intervention was then implemented beginning in Week 2.

Because of the nature of the instructional intervention, neither instructors nor students could be blinded. To reduce rating bias, skill performance was video-recorded and anonymized using coded identifiers, and three raters independently scored all performances. All three raters held national ski instructor certification and had teaching experience in university skiing courses. They underwent standardized training lasting 2 h, which included interpretation of the scoring criteria, practice scoring using example skiing videos, and procedures for resolving scoring discrepancies. After training, inter-rater consistency was evaluated using 10 pilot videos. Formal scoring began only after an intraclass correlation coefficient (ICC) of at least 0.85 had been achieved.

Under normal circumstances, the final skill score was calculated as the mean of the three ratings. If the discrepancy among the three ratings exceeded 10 points, the raters discussed the performance and reached a consensus on the final score. Psychological questionnaires were collected and entered using anonymous codes. Personnel responsible for data management and statistical analysis had no access to group information until data cleaning and model specification had been completed, thereby ensuring blinding at the statistical analysis stage.

### 2.4 Intervention

During the differential intervention phase, the two groups were matched on class duration, technical content, instructional progression, core practice tasks, and total practice volume. The only between-group difference concerned instructional interaction style and the organization of practice activities.

The autonomy-supportive teaching protocol used in the experimental group was developed based on the seven-category framework of autonomy-supportive teacher behaviors proposed by Cheon et al. (2023), with contextual adaptation for skiing instruction. The implementation strategies were as follows:

(1) Before instruction, students were asked about their perceived adaptation to situational elements such as slope gradient and speed, and instructional strategies were adjusted accordingly.
(2) After completion of core skiing tasks, optional extension exercises were provided so that students could choose within a defined range.
(3) Complex skiing movements were decomposed into progressively sequenced stepwise tasks, and informational feedback and affirmation were provided at key points.
(4) Before practice, the instructor explained the task requirements and task value, explicitly linking the movement to safe skiing and skill improvement.
(5) When frustration or fear of difficulty emerged, instructors responded using accepting language and jointly adjusted practice strategies with the students.
(6) Directive language was replaced by negotiative or invitational language to reduce controlling communication.
(7) Within the fixed class duration and task requirements, students were given limited autonomy over the number of repetitions and rest frequency. The instructor monitored performance quality throughout the process and adjusted the practice arrangement when necessary to ensure completion of the core tasks and consistency of instructional progress.

The control group maintained the conventional “demonstration–explanation–practice” instructional routine. Specifically, instructors followed the unified instructional progression by providing movement demonstrations, explaining technical points, organizing centralized practice, giving safety reminders, and offering corrective feedback. Students in the control group completed the same practice tasks according to the instructor’s arrangement. The control and experimental groups were matched in class duration, technical content, core practice tasks, and total practice volume, but the control group did not systematically implement the autonomy-supportive strategies specified in the experimental protocol, such as bounded choice, emotional acknowledgment, negotiative language, and limited autonomous arrangements.

To minimize the influence of instructor variability on between-group comparisons, the intervention was delivered by four instructors, all of whom held national ski instructor certification and had experience teaching university skiing courses. A paired allocation strategy was used such that each instructor taught one experimental class and one control class. After completion of the T0 assessments, the research team disclosed group allocation to the instructors and provided standardized training before the start of Week 2. The training lasted 3 h and covered the principles of autonomy-supportive teaching, implementation of the seven categories of autonomy-supportive behaviors, boundary conditions for conventional teaching in the control group, classroom interaction language norms, and requirements for preventing contamination between teaching conditions. After training, each instructor completed one mock teaching session. Formal intervention began only after the research team confirmed adherence to the assigned teaching protocol.

To assess intervention fidelity, the research team conducted classroom observation records during the differential intervention phase. For each class, one session in Week 2 and one session in Week 3 were observed, resulting in 16 classroom observation units. The observation records were completed by one researcher who was not involved in teaching, using a standardized checklist based on the seven autonomy-supportive teacher behaviors specified in the intervention protocol. Each item was rated on a 0–2 scale, where 0 indicated that the behavior was not observed, 1 indicated that the behavior occurred occasionally or was insufficiently implemented, and 2 indicated that the behavior was clearly observed and appropriate to the instructional context. This fidelity check was used to determine whether the experimental classes implemented the intended autonomy-supportive teaching behaviors, whether the control classes systematically displayed these behaviors, and whether the control classes maintained the conventional “demonstration–explanation–practice” instructional routine.

All instructional activities were conducted on ski slopes equipped with appropriate safety facilities. Potential risks were jointly monitored by certified instructors and professional safety personnel, and all incidents and their management outcomes were recorded according to a standardized procedure.

### 2.5 Measures

#### 2.5.1 Perceived teacher autonomy support

Perceived teacher autonomy support was assessed using the 15-item scale developed by Tilga et al. (2017). Responses were recorded on a 7-point Likert scale ranging from 1 (strongly disagree) to 7 (strongly agree). Several items were minimally adapted to the skiing context by replacing references to “physical education class” with “ski class” and “physical education teacher” with “ski instructor.” After adaptation, the scale was reviewed by three experts in sport psychology and two national-level ski instructors to confirm contextual appropriateness while preserving the original meaning of the items.

T0 data showed good internal consistency for the revised scale (Cronbach’s *α* = 0.858), supporting its use in the present sample. The final score was calculated as the mean of all items, with higher scores indicating greater perceived teacher autonomy support. In this study, this variable served as the manipulation-check indicator for the differential intervention phase. Group effects, time effects, and Group × Time interaction effects were used to evaluate whether autonomy-supportive teaching produced the intended between-group difference in perceived teacher autonomy support.

#### 2.5.2 Skill acquisition

Skill acquisition was evaluated using a pre-established standardized technical scoring rubric comprising five core dimensions: posture control (20%), center-of-mass transfer (25%), turning stability (25%), speed control (15%), and trajectory control (15%). Each dimension was rated on a 0–20 scale. A weighted composite score was then calculated by multiplying each dimensional score by its corresponding weight and converting the sum to a 0–100 scale.

Three raters with national ski instructor certification and university-level teaching experience independently scored all performances. The final score for each participant was the mean of the three ratings. Inter-rater reliability was evaluated using the intraclass correlation coefficient (ICC), based on a two-way random-effects model with absolute agreement and average measures, and the ICC with its 95% confidence interval was reported.

#### 2.5.3 Self-efficacy

Self-efficacy was measured using the General Self-Efficacy Scale (Schwarzer and Jerusalem, 1995). The scale contains 10 items rated on a 4-point scale ranging from 1 (not at all true) to 4 (exactly true). During questionnaire administration, participants were instructed to respond with reference to their current skiing course context to improve the relevance of the measure to the instructional tasks. The final score was calculated as the mean of all items, with higher scores indicating greater confidence in one’s ability to complete learning tasks. T0 data showed acceptable internal consistency for this scale in the present sample (Cronbach’s *α* = 0.795).

#### 2.5.4 Mental toughness

Mental toughness was assessed using the Sports Mental Toughness Questionnaire (SMTQ; Sheard et al., 2009). The questionnaire contains 14 items rated on a 4-point scale ranging from 1 (not at all true) to 4 (very true). The final score was calculated as the mean of all items, with higher scores indicating a stronger capacity to maintain psychological stability and sustained engagement under pressure and challenge.

The SMTQ has demonstrated good reliability and validity across multiple sport studies, with internal consistency coefficients typically ranging from 0.75 to 0.86, and its three-factor structure has been supported by confirmatory factor analysis. In the present study, T0 data showed good internal consistency (Cronbach’s *α* = 0.83), supporting its suitability for this sample.

### 2.6 Data collection procedure

Data were collected at three measurement time points (T0–T2), each scheduled at the end of the instructional sessions for the corresponding week. T0 was completed after the standardized teaching phase in Week 1, whereas T1 and T2 were completed after the instructional sessions in Weeks 2 and 3, respectively. At each time point, both skill assessment and questionnaire administration were conducted.

Skill performance was video-recorded according to a standardized procedure. The self-efficacy and mental toughness questionnaires were administered and collected by research assistants within 10 min after the end of class. All data were anonymized using coded identifiers. Questionnaire and scoring data were entered independently by two research team members and then cross-checked for accuracy.

### 2.7 Data preprocessing and statistical analysis

#### 2.7.1 Data preprocessing

Data were checked for completeness and consistency, including range checks, logical conflict checks, duplicate-record screening, and coding consistency verification. During the study, any course interruption, injury-related withdrawal, absence, or incomplete assessment was documented with respect to group assignment, time point, and reason, and then handled according to the prespecified rules.

Before model fitting, the distribution of each outcome variable was examined. Model adequacy was further evaluated using standardized residuals, outlier diagnostics, and influential-point diagnostics. Obvious data-entry errors were corrected. Observations with extreme values that were confirmed to be valid were retained, and their influence was examined further through model diagnostics and sensitivity analyses.

#### 2.7.2 Statistical analysis

All statistical analyses were conducted using SPSS 27.0. All significance tests were two-tailed, with *α* set at 0.05, and 95% confidence intervals were reported. Given the clustered class structure and repeated measurements at the individual level, perceived teacher autonomy support, skill acquisition, self-efficacy, and mental toughness were analyzed using linear mixed-effects models.

Random intercepts were specified for both class and student to account for within-cluster and within-individual correlation. Fixed effects included group (experimental vs. control), time (T0–T2), and the Group × Time interaction. The Group × Time interaction was used to test whether the two groups differed in their trajectories of change over time. Following recommendations for effect-size reporting in multilevel models, class-level ICCs were reported for the random-effects component, and standardized fixed-effect estimates were used to describe the magnitude of the main interaction effects (Lorah, 2018). Each outcome variable was further transformed into a z score, and the same linear mixed-effects model specification was refitted to estimate the standardized parameters of the Group × Time interaction terms. These standardized fixed-effect estimates were reported as standardized additional gains (βstd) in the Results section.

Linear mixed-effects model results were reported as fixed-effect estimates (b), standard errors, significance tests, and 95% confidence intervals. To describe the degree of class-level clustering, null models including only a class-level random intercept were fitted separately for the main outcomes at T0, and class-level ICCs were calculated based on variance decomposition. To examine the robustness of the results, sensitivity analyses were additionally conducted using leave-one-cluster-out re-estimation, and the direction and significance of the main conclusions were compared across models.

## 3 Results

### 3.1 Participant flow and analytic sample

A total of 228 participants were enrolled in the study (experimental group: *n* = 114; control group: *n* = 114). All eight intact class clusters completed the instructional program and assessments at T0, T1, and T2. Twelve participants (experimental group: *n* = 4; control group: *n* = 8) did not meet the prespecified attendance requirements and were excluded from the analyses. The final analytic sample comprised 216 participants (experimental group: *n* = 110; control group: *n* = 106). All exclusions were attributable to non-instructional factors, including scheduling conflicts, participation in sporting events, and seasonal illnesses such as fever or cold. No participant discontinued follow-up because of instruction-related adverse events.

### 3.2 Descriptive statistics

Table 1 presents descriptive statistics (means and standard deviations) for perceived teacher autonomy support, skill acquisition, self-efficacy, and mental toughness in the experimental and control groups at T0, T1, and T2. At T0, the two groups showed similar mean values across all four measures. Following implementation of the differential intervention (T1 and T2), raw mean scores increased in both groups, with the experimental group consistently showing higher mean values on all measures.

**Table 1:** Descriptive statistics for perceived teacher autonomy support, skill acquisition, self-efficacy, and mental toughness by group at T0, T1, and T2 (M ± SD).

Variable	Group	T0	T1	T2
Perceived Teacher Autonomy Support	Experimental	1.94 ± 0.29	6.07 ± 0.27	6.38 ± 0.27
	Control	1.98 ± 0.25	2.27 ± 0.30	2.42 ± 0.28
Skill Acquisition	Experimental	64.97 ± 6.02	77.86 ± 5.00	84.66 ± 6.05
	Control	64.62 ± 4.86	71.67 ± 3.98	76.09 ± 4.02
Self-Efficacy	Experimental	1.90 ± 0.19	2.60 ± 0.09	3.52 ± 0.12
	Control	1.91 ± 0.19	2.10 ± 0.09	2.35 ± 0.13
Mental Toughness	Experimental	2.00 ± 0.18	2.61 ± 0.15	3.51 ± 0.17
	Control	2.05 ± 0.16	2.10 ± 0.13	2.37 ± 0.13

M, mean; SD, standard deviation. Experimental group, *n*, 110; control group, *n*, 106. Skill acquisition scores ranged from 0 to 100.

### 3.3 Manipulation check: perceived teacher autonomy support

The classroom observation fidelity check results are presented in Table 2. During the differential intervention phase, the total score for the seven autonomy-supportive teaching behaviors was 12.25 ± 1.16 in the experimental group and 2.25 ± 1.04 in the control group, indicating that the experimental group implemented the intended autonomy-supportive teaching strategies to a greater extent. Although the control group showed some score on the stepwise task progression dimension, this mainly reflected movement decomposition and progressive practice commonly used in conventional skiing instruction. The control group scored lower on core autonomy-supportive dimensions, including bounded choice, emotional acknowledgment, non-controlling language, and limited autonomous arrangements, indicating that it did not systematically implement the autonomy-supportive teaching strategies specified in the experimental protocol.

**Table 2:** Classroom observation fidelity scores during the differential intervention phase (M ± SD).

Observation dimension	Experimental group	Control group
Asking about students’ adaptation	1.63 ± 0.52	0.00 ± 0.00
Providing bounded choice	1.88 ± 0.35	0.25 ± 0.46
Stepwise task progression	1.88 ± 0.35	1.00 ± 0.76
Explaining task value	1.75 ± 0.46	0.38 ± 0.52
Emotional acknowledgment	1.88 ± 0.35	0.25 ± 0.46
Non-controlling language	1.63 ± 0.52	0.25 ± 0.46
Limited autonomous arrangements	1.63 ± 0.52	0.13 ± 0.35
Total score	12.25 ± 1.16	2.25 ± 1.04

**Figure 1:**
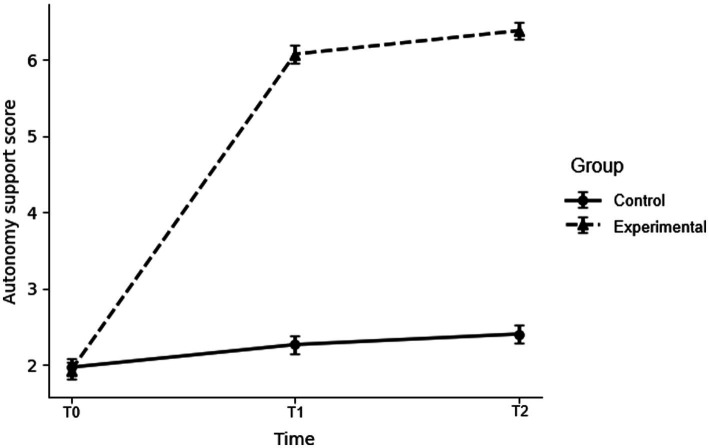
Estimated marginal mean trajectories for perceived teacher autonomy support across T0, T1, and T2.

The linear mixed-effects model for perceived teacher autonomy support further supported this judgment. The Group × Time interaction was significant, *F*(2, 279.26) = 5370.10, *p* < 0.001, indicating that the two groups differed significantly in their changes in perceived autonomy support during the differential intervention phase. No significant group difference was detected at T0 (b = −0.04, *p* = 0.566). In the control group, perceived teacher autonomy support increased by 0.30 from T0 to T1 and by 0.44 from T0 to T2 (both *p* < 0.001). Over the same intervals, the experimental group showed additional gains of 3.84 (T1–T0) and 4.00 (T2–T0) relative to the control group (both *p* < 0.001), with corresponding standardized additional gains of βstd = 1.96 and βstd = 2.04, respectively. These results indicate that autonomy-supportive teaching increased students’ perceived teacher autonomy support during the two-week differential intervention phase. The estimated marginal mean trajectories showed that perceived teacher autonomy support remained consistently higher in the experimental group during the differential intervention phase. Fixed-effect tests and parameter estimates are presented in Tables 3, 4, and the estimated marginal mean trajectories are shown in Figure 1.

**Table 3:** Tests of fixed effects for perceived teacher autonomy support.

Effect	Numerator df	Denominator df	*F*	*p*
Intercept	1	6.03	11773.56	*p* < 0.001
Group	1	6.03	1579.56	*p* < 0.001
Time	2	279.26	7612.90	*p* < 0.001
Group × Time	2	279.26	5370.10	*p* < 0.001

Dependent variable: perceived teacher autonomy support.

**Table 4:** Fixed-effect parameter estimates for perceived teacher autonomy support.

Parameter	Estimate	SE	df	*t*	*p*	95% CI	
						Lower	Upper
Intercept	1.98	0.05	8.01	40.25	p < 0.001	1.86	2.09
Experimental vs. control (T0)	−0.04	0.07	7.93	−0.60	p = 0.566	−0.20	0.12
Control: T1–T0	0.30	0.03	258.23	9.54	p < 0.001	0.23	0.36
Control: T2–T0	0.44	0.03	304.98	14.05	p < 0.001	0.38	0.51
Interaction: experimental additional gain (T1–T0)	3.84	0.04	258.23	88.49	p < 0.001	3.75	3.92
Interaction: experimental additional gain (T2–T0)	4.00	0.04	304.98	90.41	p < 0.001	3.91	4.09

Reference categories: control group and T0.

### 3.4 Primary outcome: skill acquisition

**Figure 2:**
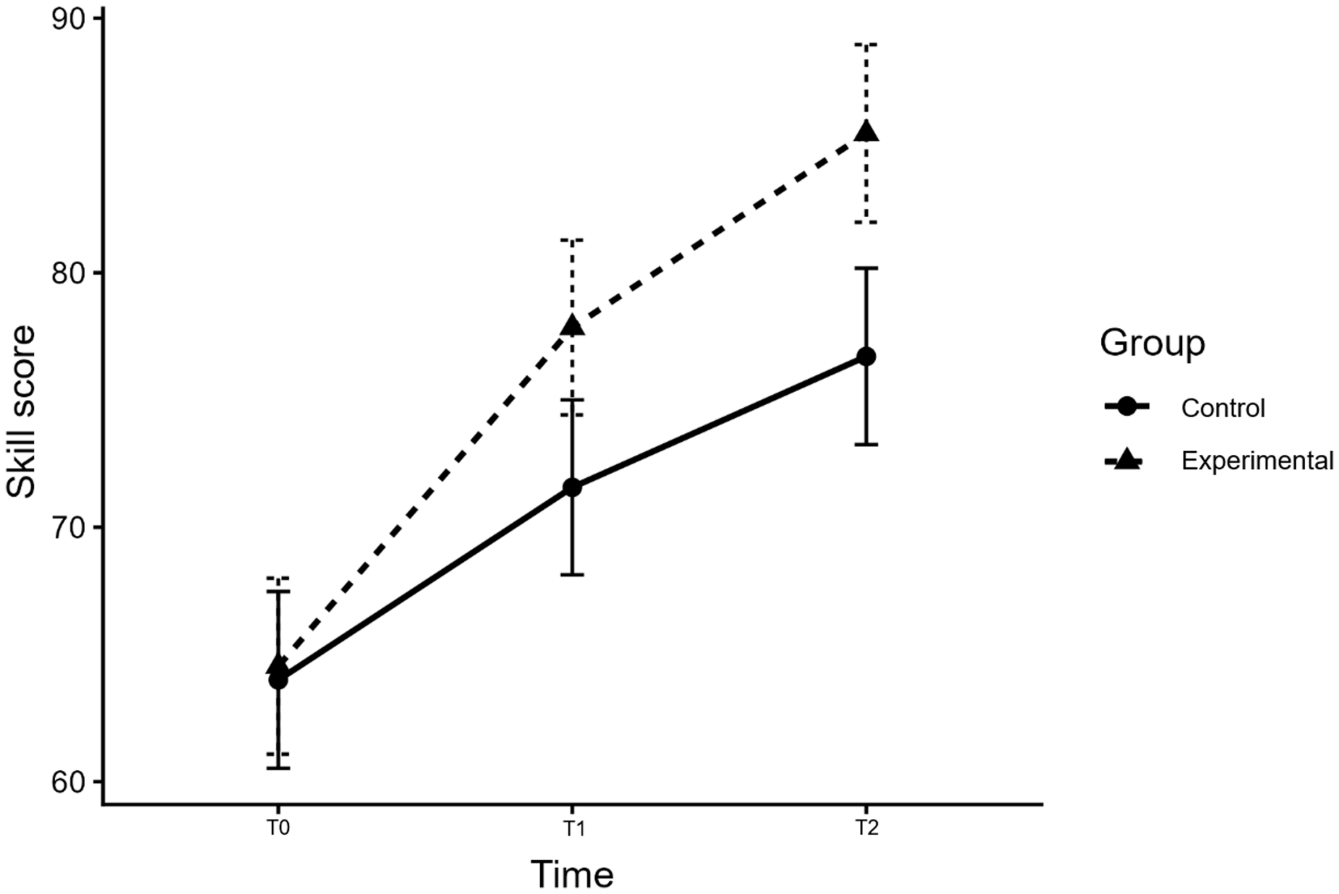
Estimated marginal mean trajectories for skill acquisition across T0, T1, and T2 by group.

Linear mixed-effects model analysis revealed a significant Group × Time interaction, *F*(2, 248.12) = 108.54, *p* < 0.001, indicating that the two groups differed significantly in their trajectories of skill acquisition over time. No significant group difference was detected at T0 (b = 0.41, *p* = 0.839). In the control group, skill scores increased by 7.05 from T0 to T1 and by 11.47 from T0 to T2 (both *p* < 0.001), suggesting that conventional teaching and repeated practice also promoted skill improvement among novice skiers. Over the same intervals, the experimental group showed additional gains of 5.84 points (T1–T0) and 8.22 points (T2–T0) relative to the control group (both *p* < 0.001), with corresponding standardized additional gains of βstd = 0.67 and βstd = 0.94, respectively. Because skill acquisition was scored on a 0–100 scale, these values indicate that autonomy-supportive teaching produced an educationally meaningful improvement in skill performance without increasing instructional time or practice volume. In terms of the scoring dimensions, this improvement reflected further development in integrated technical performance, including posture control, center-of-mass transfer, turning stability, speed control, and trajectory control. The estimated marginal mean trajectories showed that both groups improved over time; however, the experimental group showed higher estimated means at T1 and T2, and the between-group difference widened over time. Inter-rater reliability for skill scores was high (ICC = 0.89, 95% CI [0.84, 0.93]). Fixed-effect tests and parameter estimates are presented in Tables 5, 6, and the estimated marginal mean trajectories are shown in Figure 2.

**Table 5:** Tests of fixed effects for skill acquisition.

Effect	Numerator df	Denominator df	*F*	*p*
Intercept	1	5.99	5806.08	*p* < 0.001
Group	1	5.99	7.02	*p* = 0.038
Time	2	248.12	1517.64	*p* < 0.001
Group × Time	2	248.12	108.54	*p* < 0.001

Dependent variable: skill acquisition.

**Table 6:** Fixed-effect parameter estimates for skill acquisition.

Parameter	Estimate	SE	df	*t*	*p*	95% CI	
						Lower	Upper
Intercept	64.63	1.38	6.42	46.68	*p* < 0.001	61.30	67.97
Experimental vs. control (T0)	0.41	1.96	6.39	0.21	*p* = 0.839	−4.30	5.13
Control: T1–T0	7.05	0.41	254.73	17.36	*p* < 0.001	6.25	7.85
Control: T2–T0	11.47	0.41	217.61	28.20	*p* < 0.001	10.67	12.27
Interaction: experimental additional gain (T1–T0)	5.84	0.57	254.73	10.27	*p* < 0.001	4.72	6.96
Interaction: experimental additional gain (T2–T0)	8.22	0.57	217.61	14.42	*p* < 0.001	7.10	9.34

Reference categories: control group and T0.

The class-level ICC for skill acquisition at T0 was 0.283, indicating a degree of class-level clustering in skill scores and supporting the inclusion of a class-level random intercept in the linear mixed-effects models. Sensitivity analyses using a leave-one-cluster-out approach showed that the direction of the Group × Time interaction remained unchanged, and the inferential conclusions were consistent with the primary analysis.

### 3.5 Secondary outcome: self-efficacy

**Figure 3:**
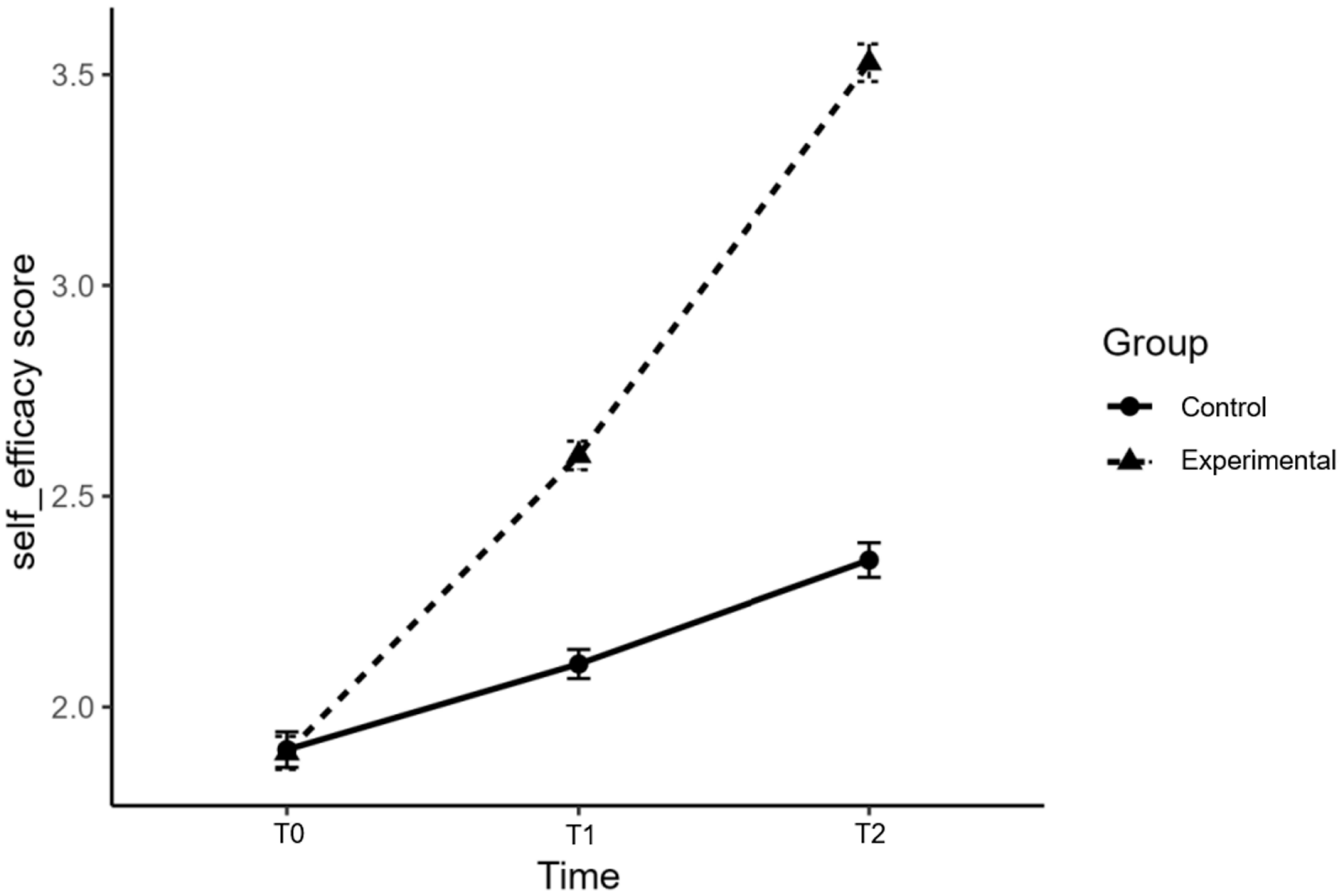
Estimated marginal mean trajectories for self-efficacy across T0, T1, and T2 by group.

Linear mixed-effects model analysis revealed a significant Group × Time interaction, *F*(2, 242.48) = 1240.60, *p* < 0.001, indicating that the two groups differed significantly in their trajectories of self-efficacy over time. No significant group difference was detected at T0 (b = −0.01, *p* = 0.850). In the control group, self-efficacy increased by 0.19 from T0 to T1 and by 0.44 from T0 to T2 (both *p* < 0.001), suggesting that conventional skiing instruction also produced some improvement in self-efficacy. Over the same intervals, the experimental group showed additional gains of 0.50 (T1–T0) and 1.18 (T2–T0) relative to the control group (both *p* < 0.001), with corresponding standardized additional gains of βstd = 0.86 and βstd = 2.03, respectively. These results indicate that, after the 2-week differential intervention, learners in the experimental group showed a more pronounced improvement in their perceived capability to complete skiing learning tasks than those in the control group. The estimated marginal mean trajectories showed a steeper increase in self-efficacy in the experimental group during the differential intervention period. Fixed-effect tests and parameter estimates are presented in Tables 7, 8, and the estimated marginal mean trajectories are shown in Figure 3.

**Table 7:** Tests of fixed effects for self-efficacy.

Effect	Numerator df	Denominator df	*F*	*p*
Intercept	1	7.29	53042.93	*p* < 0.001
Group	1	7.29	716.98	*p* < 0.001
Time	2	242.48	3744.09	*p* < 0.001
Group × Time	2	242.48	1240.60	*p* < 0.001

Dependent variable: self-efficacy.

**Table 8:** Fixed-effect parameter estimates for self-efficacy.

Parameter	Estimate	SE	df	*t*	*p*	95% CI	
						Lower	Upper
Intercept	1.91	0.02	29.51	90.43	*p* < 0.001	1.86	1.95
Experimental vs. control (T0)	−0.01	0.03	28.81	−0.19	*p* = 0.85	−0.07	0.05
Control: T1–T0	0.19	0.02	235.64	10.77	*p* < 0.001	0.16	0.23
Control: T2–T0	0.44	0.02	301.95	22.69	*p* < 0.001	0.40	0.48
Interaction: experimental additional gain (T1–T0)	0.50	0.02	235.64	20.36	*p* < 0.001	0.46	0.55
Interaction: experimental additional gain (T2–T0)	1.18	0.03	301.95	43.67	*p* < 0.001	1.13	1.24

Reference categories: control group and T0.

The class-level ICC for self-efficacy at T0 was 0.070, indicating weak class-level clustering. Sensitivity analyses using a leave-one-cluster-out approach showed that the direction of the Group × Time interaction remained unchanged, and the inferential conclusions were consistent with the primary analysis.

### 3.6 Secondary outcome: mental toughness

**Figure 4:**
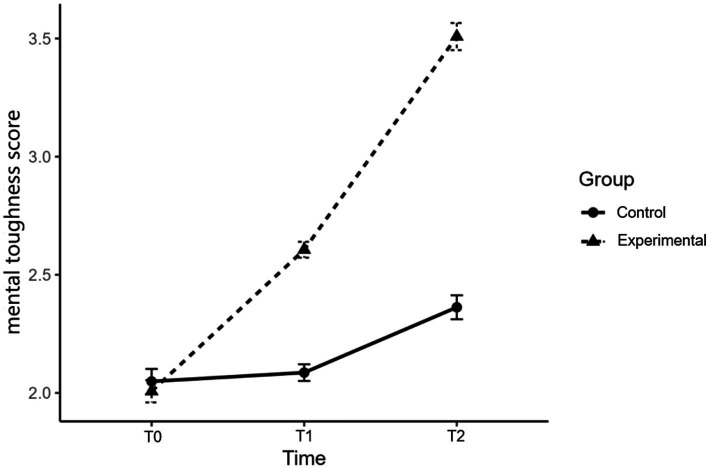
Estimated marginal mean trajectories for mental toughness across T0, T1, and T2 by group.

Linear mixed-effects model analysis revealed a significant Group × Time interaction, *F*(2, 260.02) = 1035.43, *p* < 0.001, indicating that the two groups differed significantly in their trajectories of mental toughness over time. No significant group difference was detected at T0 (b = −0.05, *p* = 0.068). In the control group, mental toughness increased by 0.05 from T0 to T1 (*p* = 0.013) and by 0.32 from T0 to T2 (*p* < 0.001). Over the same intervals, the experimental group showed additional gains of 0.56 (T1–T0) and 1.19 (T2–T0) relative to the control group (both *p* < 0.001), with corresponding standardized additional gains of βstd = 1.02 and βstd = 2.17, respectively. Given that mental toughness was measured on a 4-point scale, the 1.19-point additional gain from T0 to T2 indicates that, after the 2-week differential intervention, learners in the experimental group reported greater sustained engagement and pressure-coping capacity when facing learning tasks than those in the control group. The estimated marginal mean trajectories showed a steeper increase in mental toughness in the experimental group during the differential intervention period. Fixed-effect tests and parameter estimates are presented in Tables 9, 10, and the estimated marginal mean trajectories are shown in Figure 4.

**Table 9:** Tests of fixed effects for mental toughness.

Effect	Numerator df	Denominator df	*F*	*p*
Intercept	1	6.46	87528.71	*p* < 0.001
Group	1	6.46	1057.66	*p* < 0.001
Time	2	260.02	2633.71	*p* < 0.001
Group × Time	2	260.02	1035.43	*p* < 0.001

Dependent variable: mental toughness.

**Table 10:** Fixed-effect parameter estimates for mental toughness.

Parameter	Estimate	SE	df	*t*	*p*	95% CI	
						Lower	Upper
Intercept	2.05	0.02	26.97	120.10	*p* < 0.001	2.02	2.09
Experimental vs. control (T0)	−0.05	0.02	26.14	−1.90	*p* = 0.068	−0.09	0.003
Control: T1–T0	0.05	0.02	286.07	2.51	*p* = 0.013	0.01	0.08
Control: T2–T0	0.32	0.02	285.58	17.21	*p* < 0.001	0.29	0.36
Interaction: experimental additional gain (T1–T0)	0.56	0.03	286.07	22.03	*p* < 0.001	0.51	0.61
Interaction: experimental additional gain (T2–T0)	1.19	0.03	285.58	45.05	*p* < 0.001	1.13	1.24

Reference categories: control group and T0.

The class-level ICC for mental toughness at T0 was 0.019, indicating low class-level clustering. Sensitivity analyses using a leave-one-cluster-out approach showed that the direction of the Group × Time interaction remained unchanged, and the inferential conclusions were consistent with the primary analysis.

## 4 Discussion

### 4.1 Main findings and hypothesis testing

Using a cluster-randomized controlled design in the context of beginner skiing instruction, the present study showed that the two groups formed distinguishable instructional environments during the differential intervention phase, as evidenced by the manipulation check. Learners in the experimental group reported significantly greater increases in perceived teacher autonomy support than those in the control group, indicating that the intended between-group difference in perceived teacher autonomy support was achieved. Further analyses showed significant Group × Time interaction effects for skill acquisition, self-efficacy, and mental toughness. In terms of raw-scale changes from T0 to T2, the experimental group showed additional gains relative to the control group of 8.22 points in skill acquisition, 1.18 points in self-efficacy, and 1.19 points in mental toughness. The standardized additional gains further supported this pattern. These findings indicate that autonomy-supportive teaching produced educationally and practically meaningful improvements in skill performance and psychological outcomes within a short-cycle beginner skiing program.

Given the relatively large short-term increases in self-efficacy, mental toughness, and perceived teacher autonomy support, these findings should be interpreted cautiously in relation to the instructional context. Although the differential intervention lasted only 2 weeks, students received three 200-min sessions per week, resulting in a concentrated learning schedule. For novice skiers, this high-frequency and continuous learning process may rapidly increase familiarity with movement tasks and practice routines. At the same time, novice skiers face salient risk perceptions related to speed control, slope variation, falling experiences, and fear. Under these conditions, autonomy-supportive teaching may more readily influence learners’ immediate evaluations of self-efficacy and mental toughness. Therefore, the increases in self-efficacy and mental toughness are better understood as short-term improvements in self-reported psychological resources within the instructional context, rather than as enduring changes in stable psychological traits. Overall, the findings are consistent with existing evidence that autonomy-supportive teaching can improve the quality of learning engagement, motivational processes, and adaptive learning outcomes (Soe et al., 2025; Behzadnia et al., 2025). The present study extends this evidence from general classroom and conventional physical education contexts to a high-risk, short-cycle instructional context, providing longitudinal evidence with greater contextual relevance for pedagogical intervention research in skiing education.

With respect to H1, the skill acquisition findings showed no significant between-group difference at baseline, indicating that the two groups started from a comparable performance level. As instruction progressed, the control group improved significantly at both T1 and T2, suggesting that conventional teaching combined with repeated practice can facilitate skill development in beginners. However, over the same time intervals, the experimental group achieved significantly greater incremental gains, and the between-group gap widened over time. This pattern not only supports the proposition that autonomy-supportive teaching is associated with greater skill improvement, but also suggests that, even when instructional content, pacing, and total practice volume are held constant, classroom interaction style may still shape the rate of short-term skill development by influencing the quality of practice engagement, persistence, and error-correction efficiency (Cheon et al., 2022; Wang et al., 2025).

With respect to H2, self-efficacy also showed a significant Group × Time interaction. The two groups began from comparable baseline levels; although self-efficacy increased in the control group over time, the increase was substantially greater in the experimental group. This result is consistent with social cognitive theory and with instructional research emphasizing the roles of mastery experiences, informational feedback, and task framing in the development of self-efficacy (Miao and Ma, 2023; Guo et al., 2025; Li and Zeng, 2025). In beginner skiing, learning tasks are characterized by substantial technical difficulty and heightened risk perception. Learners’ judgments about whether they can execute a movement successfully or maintain stable control of speed and posture are therefore especially vulnerable to failure experiences and emotional reactions. By modifying task explanation, feedback delivery, and emotional responsiveness, autonomy-supportive teaching may strengthen learners’ perceptions of task attainability, increase the salience of mastery experiences, and reduce threat-based interpretations of setbacks, thereby contributing to faster growth in self-efficacy.

With respect to H3, mental toughness likewise showed a significant Group × Time interaction. No significant between-group difference was observed at baseline; although the control group showed some improvement over time, the experimental group demonstrated greater gains. This pattern has particular theoretical relevance in high-risk skill-learning contexts. Beginner skiing is often accompanied by falls, tension, frustration, and fluctuating risk appraisal. Under such conditions, controlling instructional language and high-pressure error correction may amplify threat perception and elicit avoidance responses. By contrast, autonomy-supportive interaction places greater emphasis on emotional acknowledgment, rationale provision, and negotiated guidance, all of which may help learners sustain goal-directed behavior and remain engaged under pressure (Tunca, 2024). In this respect, the present findings provide empirical evidence relevant to the cultivation of psychological resources within high-risk sport instruction.

### 4.2 Potential mechanisms: an SDT-based interpretation

The experimental group showed greater time-related gains in skill acquisition, self-efficacy, and mental toughness. This pattern is consistent with the Self-Determination Theory explanation that supportive teacher interactions are associated with more adaptive learning outcomes (Ryan and Deci, 2020).

From the perspective of autonomy support, the greater gains observed in the experimental group may be related to “bounded choice within clear boundaries.” Beginner skiing involves explicit safety constraints and staged technical progression. In this context, autonomy support does not mean weakening structure or lowering standards. Rather, it involves providing limited choice, rationale provision, and opportunities for negotiation within fixed task goals, instructional pacing, and safety boundaries. This type of teaching may help learners experience practice as an engaging and adjustable challenge, thereby reducing avoidance tendencies (Haerens et al., 2015). This interpretation is also consistent with evidence showing that teacher autonomy support is trainable and that changes in teaching behavior can reliably improve classroom experience (Tilga and Koka, 2025).

From the perspective of competence support, the concurrent advantages observed in skill scores and self-efficacy may be related to learners obtaining clearer and more noticeable experiences of movement progress. Hierarchical task design, informational feedback, and explicit cues about progress may help learners interpret errors as modifiable process information. During the beginner stage, this attributional orientation is especially important for sustaining repeated attempts and continued practice. Research in physical education has shown that teacher need-supportive behaviors can influence students’ classroom engagement and behavioral outcomes through psychological need satisfaction (Vasconcellos et al., 2020; Tilga and Koka, 2025). In the present context, the parallel increases in skill acquisition and self-efficacy are consistent with the theoretical explanation that clearer perception of movement progress may strengthen task confidence and support continued practice engagement. However, this pathway should be examined in future studies using mediation models.

From the perspective of relatedness support, the greater improvement in mental toughness observed in the experimental group may be related to teachers’ accepting responses to fear, frustration, and uncertainty. Beginner skiers are more likely to experience threat appraisal and defensive reactions when facing speed, slope variation, and the risk of falling. When teachers respond to these experiences in a non-controlling manner, provide rationales, and negotiate adjustments to practice strategies, learners may be more likely to maintain goal-directed behavior. Recent classroom research has reported significant associations between teachers’ motivating styles and students’ mental toughness and grit, and has identified psychological need satisfaction as a mediating mechanism (Cheon et al., 2024). The similar pattern observed in the present high-risk skill-learning context is consistent with the view that need support and psychological resource enhancement may help explain intervention effects, although this pathway requires direct testing in future research.

### 4.3 Practical implications for high-risk skill instruction

The practical significance of this study does not lie in increasing instructional time, expanding practice volume, or altering technical content per se. Its central contribution is to show that, within a standardized instructional structure and fixed safety boundaries, optimizing classroom interaction style can simultaneously improve skill performance and key psychological resources. Given the larger time-related gains observed in the experimental group across skill acquisition, self-efficacy, and mental toughness, autonomy-supportive teaching can be understood in practice as an organizational approach for improving learning quality under highly structured conditions, rather than as a weakening of instructional control. This interpretation is consistent with the broader SDT-based literature in physical education, which has shown robust positive associations between need-supportive teaching and more adaptive motivation, participation, and learning outcomes (Vasconcellos et al., 2020). More recent evidence further suggests that combining autonomy support with structure support is more beneficial than emphasizing either dimension alone (Patzak and Zhang, 2025).

At the level of instructional organization, a feasible strategy is to design tasks with clear goals, explicit boundaries, and limited choice. High-risk skill instruction necessarily requires unified pacing, stable safety rules, and a consistent core practice volume. Even so, bounded choice can still be incorporated—for example, by offering practice pathways with different difficulty gradients under the same technical theme, providing optional extension tasks after completion of core requirements, or allowing learners to adjust repetition frequency and rest intervals within predefined limits. Such arrangements do not weaken the teacher’s responsibility for risk management. Instead, they may strengthen learners’ sense of controllability and improve the quality of task engagement, making practice more likely to be experienced as an adjustable challenge rather than passive execution (Tilga and Koka, 2025).

At the level of feedback and communication, pedagogical refinement should focus on three closely related elements: informational feedback, non-controlling language, and emotional responsiveness. For beginner skiers, performance setbacks and risk perception are tightly intertwined. When feedback relies heavily on commands, evaluative negation, or decontextualized error correction, learners may be more likely to develop threat appraisals and avoidance tendencies. By contrast, organizing feedback around key movement cues, actionable correction pathways, and renewed opportunities for practice is more likely to preserve competence experience and support persistence. At the same time, accepting responses to fear, tension, and frustration should not be viewed merely as optional climate management. Rather, they should be integrated into safe instruction itself, because emotional stability directly influences movement control and risk-coping quality (Zhao et al., 2023).

At the level of teacher training and course management, the present findings suggest that autonomy-supportive teaching has strong potential for standardized implementation. Instructional improvement does not necessarily require long-cycle or resource-intensive reform. A more feasible approach may be short-cycle teacher training focused on specific, observable, teachable, and feedback-sensitive behavioral units, such as how to provide bounded choice, how to explain task value, how to use non-controlling language templates, how to respond to learner emotions, and how to define the boundaries of classroom negotiation. Recent work in physical education has shown that need-supportive teacher training, including online formats, can improve teachers’ autonomy-supportive and competence-supportive behaviors as well as their teaching efficacy, thereby offering a realistic pathway for large-scale teacher development in universities and training institutions (Tilga and Koka, 2025).

### 4.4 Strengths and limitations

This study employed a cluster-randomized controlled design in a high-risk skill-learning context of beginner skiing and repeatedly measured objective skill performance and key psychological variables within the same instructional cycle. This design allowed the short-term effects of autonomy-supportive teaching to be examined under authentic classroom conditions. The manipulation check showed that the two groups were clearly differentiated in learners’ perceived teacher autonomy support during the differential intervention phase, strengthening the interpretation of the source of differences in the main outcomes. Combined with cluster randomization and longitudinal modeling, the present study provides context-specific empirical support for the application of autonomy-supportive teaching in ice and snow sport education.

In terms of strengths, first, the study used a cluster-randomized design and linear mixed-effects models to account for class-level clustering and repeated measurements, thereby better reflecting the organizational structure of real teaching and improving ecological validity. Second, the outcome indicators covered both objective behavioral performance and psychological resource variables, avoiding the separation of skill improvement from psychological change and allowing a more comprehensive evaluation of the intervention. Third, skill assessment was based on video recordings, independent ratings by multiple raters, and formal reporting of inter-rater reliability, which reduced the risk of scoring bias associated with real-time evaluation and instructor expectations. Fourth, the intervention protocol was derived from an established theoretical framework and adapted to the skiing task context, making the implementation content clearly describable, reproducible, and suitable for further testing.

Several limitations should be acknowledged. First, although classroom observation records and the manipulation check were used to monitor intervention implementation, the classroom observations were completed by a single observer. Therefore, the objective assessment of autonomy-supportive teaching behaviors remained limited. Second, although skill ratings and questionnaires were anonymized, psychological variables and perceived teacher autonomy support may still have been influenced by expectancy effects, response bias, and common method bias. In addition, the study used a general self-efficacy scale. Although this scale can reflect learners’ overall task confidence, it does not directly cover skiing-specific tasks such as speed control, slope adaptation, turn completion, or continued practice after falling. Therefore, it may be less sensitive than a skiing-specific self-efficacy measure. Third, the intervention period was short, and the findings mainly reflect short-term changes during the beginner stage. In particular, the rapid increases in self-efficacy and mental toughness are better interpreted as short-term improvements in self-reported psychological resources, which require further verification through longer follow-up and multi-method assessment. Fourth, the participants were novice skiers from a sport university. Because they had a certain level of physical foundation and prior adaptation to sport courses, the findings may not be directly generalizable to all novice skier populations. Fifth, the number of clusters in the cluster-randomized trial was relatively limited, and unmeasured class-level factors may still have influenced estimation precision.

### 4.5 Future directions

Building on the short-term intervention evidence provided here, future research should extend this line of inquiry in four directions to strengthen the robustness and applied value of the conclusions.

First, future studies should increase the number of clusters and conduct replication across multiple contexts. Larger cluster-randomized trials conducted across different schools, ski-field conditions, and instructional teams would improve the precision of class-level inference and help clarify the boundary conditions associated with peer climate, class culture, and teacher variability. Stratified replication across different learning stages would also be valuable for comparing the magnitude and developmental trajectories of autonomy-supportive teaching effects at beginner versus more advanced stages.

Second, future studies should incorporate mechanism variables and test mediation pathways directly. Within the SDT framework, variables such as psychological need satisfaction and frustration, motivational regulation, emotion regulation, threat appraisal, and self-regulation of practice could be introduced as process indicators. Longitudinal mediation models or multilevel structural equation models could then be used to test the proposed sequence linking autonomy support to need-related processes, motivational quality and self-regulation, behavioral engagement, and ultimately skill and psychological outcomes. Such designs would reduce reliance on theoretical inference and help identify the most consequential mediating links and intervention targets.

Third, future work should extend follow-up periods and examine retention, transfer, and safety-related outcomes. Additional follow-up assessments would make it possible to evaluate skill retention, cross-task transfer, and stability across ski seasons. At the same time, safety-related outcomes should be incorporated more systematically, including falls and near-miss incidents, risk-taking behavior, avoidance tendencies, and training adherence, thereby providing a more comprehensive account of both the benefits and potential costs of high-risk skill instruction.

Fourth, future studies should optimize the measurement system and strengthen the multi-method evidence base. In addition to self-report scales, behavioral and physiological indicators could be incorporated to reduce common-method bias, including objective records of practice quantity and quality, observational indices of classroom engagement, heart-rate variability or other stress-related indicators, and video-based quantification of movement stability. For self-efficacy and mental toughness, future work could also compare the sensitivity of general versus context-specific measures in detecting teaching-related change.

Overall, future research should combine larger samples, longer follow-up periods, mechanism variables, and multi-method assessment strategies to clarify more precisely the operative pathways, external validity, and implementation conditions of autonomy-supportive teaching in high-risk skill learning environments.

## Data Availability

The raw data supporting the conclusions of this article will be made available by the authors, without undue reservation.
